# The role of stigma as an obstacle for social inclusion for people with severe mental disorders

**DOI:** 10.1192/j.eurpsy.2023.128

**Published:** 2023-07-19

**Authors:** N. Sartorius

**Affiliations:** Association for the Improvement of Mental Health Programmes (AMH), Geneva, Switzerland

## Abstract

**Abstract:**

Stigma continues to be the main obstacle to the improvement of mental health care and to a life of good quality for people with mental disorders or with the experience of a mental disorder. It affects all that is related to mental illness, not only the person who has the disorder but also the institutions in which people with mental disorders receive treatment, treatment means, such as medications, staff working in mental health care and the family of the person with mental disorder.

Recent years have witnessed effective programs against stigma in various countries and it would be logical to expect that work against stigma will be a crucial part of mental health programs. This unfortunately is not the case.

The presentation will focus on interventions that have been successful in reducing stigmatisation or its consequences and propose action to reduce stigma.

**Disclosure of Interest:**

None Declared

